# Household Disposal of Unused and Expired Medications: A Cross‐Sectional Study on Awareness, Attitude, and Practice in Boma Health Zone, Democratic Republic of Congo, 2024

**DOI:** 10.1155/jt/4699974

**Published:** 2026-01-29

**Authors:** Christian M. Valuvunina, Guillaume M. Kiyombo, Joël Nkiama N. Konde

**Affiliations:** ^1^ Department of Environmental Health, School of Public Health, University of Kinshasa, Kinshasa, Democratic Republic of the Congo, unikin.ac.cd; ^2^ School of Public Health, University of Kinshasa, Kinshasa, Democratic Republic of the Congo, unikin.ac.cd

**Keywords:** attitude, awareness, democratic Republic of Congo, household disposal, pharmaceutical waste, practice, public health, unused and expired medication

## Abstract

**Background:**

The accumulation of unused and expired medications (UEM) in households represents a growing public health and environmental concern, particularly in low‐resource settings such as the Democratic Republic of Congo (DRC), where safe disposal infrastructure is limited and regulatory frameworks are weak. In the Boma Health Zone, previous environmental studies have documented pollution of the Kalamu River by solid and liquid waste, including pharmaceuticals, altering its physico‐chemical and bacteriological properties. However, no systems for safe medication disposal are available to the public, and empirical data on household UEM disposal practices in the DRC are virtually nonexistent, highlighting the critical need for this research.

**Objective:**

This study assessed the knowledge, attitude, and practice (KAP) regarding UEM disposal among households in the Boma Health Zone, DRC.

**Methods:**

A community‐based cross‐sectional survey was conducted in April 2024 among 384 households, selected using a four‐stage random sampling technique. Data were collected through face‐to‐face interviews using a structured questionnaire adapted from validated KAP surveys and administered via the Open Data Kit (ODK) app. Reliability was assessed using Cronbach’s alpha (*α* = 0.78). Descriptive statistics and cross‐tabulations were performed using STATA version 14. Missing data were checked at entry via built‐in ODK validations, and incomplete questionnaires were excluded from analysis.

**Results:**

More than half of households (53.4%, *n* = 205) stored UEM, primarily due to symptom resolution (70.6%, *n* = 271). Awareness of safe disposal was poor: only 12.8% (*n* = 49) had received prior information, and 94.0% (*n* = 361) were unaware of take‐back systems. Overall, 72.9% (*n* = 276) had low awareness scores. Attitudes were more favorable, with 53.4% (*n* = 205) displaying a positive attitude and a majority (53.5%, *n* = 207) supporting mandatory take‐back programs. However, unsafe practices dominated: the most common methods for disposing of expired medications were burning (41.7%, *n* = 160) and disposal in household waste (32.8%, *n* = 126). Only 4.4% (*n* = 17) returned expired medicines to a pharmacy, resulting in 98.7% (*n* = 379) being classified as having poor disposal practices.

**Conclusion:**

Critical gaps in awareness and practice regarding UEM disposal persist in Boma, despite a willingness to engage in safer practices. Urgent, multilevel interventions are needed, including community awareness campaigns, the establishment of accessible take‐back programs, and the development of a national pharmaceutical waste management policy.

## 1. Introduction

The benefits of increased global pharmaceutical consumption are undeniable, yet this rise has led to a growing public health and environmental concern: the accumulation of unused and expired medications (UEM) within households Inappropriate disposal methods, such as burning, flushing, or discarding UEM into household waste, introduce active pharmaceutical ingredients into soil and water systems, contributing to ecotoxicological effects and the development of antimicrobial resistance (AMR) [1–3]. The World Health Organization (WHO) estimates that a substantial proportion of medicines worldwide are prescribed, dispensed, or used inappropriately, exacerbating this issue [4, 5]. Studies across diverse settings confirm that unsafe disposal remains a common household practice [6–9].

This challenge is particularly acute in low‐resource settings of sub‐Saharan Africa, where formal waste management systems and pharmaceutical take‐back programs are scarce or nonexistent [10–14]. In Ghana and Nigeria, over 70% of households report disposing of unused medicines in household trash [10, 11]. In the Democratic Republic of Congo (DRC), weak infrastructure, limited public awareness, and an absent regulatory framework for household pharmaceutical waste create a perfect storm for environmental contamination and public health risks. The Boma Health Zone (HZ), located in Kongo Central Province and serving a dense urban population, exemplifies this problem. Previous environmental studies in Boma have documented the pollution of the Kalamu River by solid and liquid waste, including pharmaceuticals, altering its physicochemical and bacteriological properties [15, 16]. Despite this, no systems for safe medication disposal are in place for the public, and no published studies have assessed the extent of this public health issue in the DRC.

Globally, there is growing recognition of the problem, but empirical evidence on household UEM disposal practices in the DRC is virtually nonexistent. Data on community awareness, attitudes, and practices (KAP) are critical for designing effective, culturally appropriate public health interventions. This study therefore aims to assess KAP regarding UEM disposal in the Boma HZ. The findings will provide essential baseline evidence to guide local awareness campaigns, inform the design of take‐back systems, and advocate for the development of national pharmaceutical waste management policies.

## 2. Methods

A community‐based cross‐sectional study was conducted in April 2024 in the Boma HZ, an administrative unit in Kongo Central Province, DRC. The HZ serves an estimated population of over 527,000 residents across 11 health areas [17, 18].

Eligible participants were adults (≥ 18 years) who were household heads or representatives residing in the Boma HZ for at least one year. Individuals with severe cognitive impairments or who declined consent were excluded. The sample size was calculated at 384 using the Cochran formula for a finite population (*N* = 527,000), assuming a conservative proportion (*p*) of 50%, a margin of error of 5%, and a 95% confidence level. A total of 387 households were enrolled to account for potential non‐response, but the final analyzed sample was 384, with incomplete records excluded. A four‐stage probability sampling technique was applied: (1) random selection of 5 out of 11 health areas; (2) random selection of 5 streets per selected area; (3) systematic sampling of 15 compounds (plots) per street; and (4) random selection of one household per compound. In this study, a “compound/plot” refers to a residential parcel that may include one or more households.

A structured questionnaire was developed based on previously validated KAP surveys [12, 13]. It was translated into Kikongo and Lingala, back‐translated into French to ensure accuracy, and pretested in Boma Bungu HZ. The tool included four sections: (A) sociodemographic data, (B) awareness (5 questions), (C) attitude (6 Likert‐scale items), and (D) practice (3 questions). The scoring systems are summarized in Table 1. Internal consistency of the attitude and practice sections was assessed using Cronbach’s alpha (*α* = 0.78), confirming acceptable reliability.

**TABLE 1:** Scoring systems for awareness, attitude, and practice.

Domain	No. of items	Scoring method and response values	Classification
Awareness	5	1 point per correct answer; 0 for incorrect/don’t know. Total score: 0–5	Low (0–2), Average (3), Good (4–5)
Attitude	6	Likert‐scale: “Strongly Agree” = +2, “Agree” = +1, “Neutral” = 0, “Disagree” = −1, “Strongly Disagree” = ‐2. Total score range: −12 to +12	Negative (< 6), Positive (≥ 6)
Practice	3	Items scored from −2 to +2 based on safety. Total score range: −6 to +6	Poor (0–3), Good (4–6)

Ten data collectors were trained for 2 days on study objectives, sampling, ethics, and use of the Open Data Kit (ODK) app. Data were collected electronically on tablets with built‐in validation rules to minimize errors and enforce completeness. Incomplete records were excluded from the analysis.

Data were exported from the ODK server to STATA version 14. Descriptive statistics (frequencies, percentages, medians, and interquartile ranges) summarized participant characteristics and KAP levels. Cross‐tabulations were performed to explore associations between independent and outcome variables. Since the study was primarily descriptive, inferential tests are reserved for a subsequent publication.

Ethical approval was granted by the Kinshasa School of Public Health’s Ethics Committee and the administrative authorities of Boma City. Written informed consent was obtained from all participants. Data confidentiality and anonymity were maintained throughout the study [19].

## 3. Results

### 3.1. Sociodemographic Characteristics

The study included 384 participants (138 men; 246 women) aged 18–88 years. The median age was 37 years (IQR: 29–47), and the majority were female (64.1%, *n* = 246). Most participants had completed secondary education (38.8%, *n* = 149) and were married (60.2%, *n* = 231). Detailed sociodemographic characteristics are presented in Table 2.

**TABLE 2:** Sociodemographic characteristics of respondents (*n* = 384).

Characteristics	*n*	%
Median age (IQR)		37 (29–47)
Relationship to Household Head		
Head	194	50.5
Head’s spouse	152	39.6
Other	38	9.9
Gender		
Female	246	64.1
Male	138	35.9
Religion		
Revival Church	144	37.5
Catholic	93	24.2
Protestant	91	23.7
Kimbanguist	23	6.0
Muslim	7	1.8
Other	26	6.8
Educational level		
None	19	4.9
Primary unfinished	29	7.6
Primary completed	29	7.6
Secondary unfinished	75	19.5
Secondary completed	149	38.8
University	83	21.6
Marital status		
Married	231	60.2
Single	107	27.9
widowed	29	7.6
Divorced/separated	17	4.4
Occupation		
Self‐employed	149	38.8
Homemaker	105	27.3
Civil servant	41	10.7
Student	38	9.9
Unemployed	36	9.4
Retired	8	2.1
Other	7	1.8

### 3.2. Awareness of Medications Disposal

**FIGURE 1:**
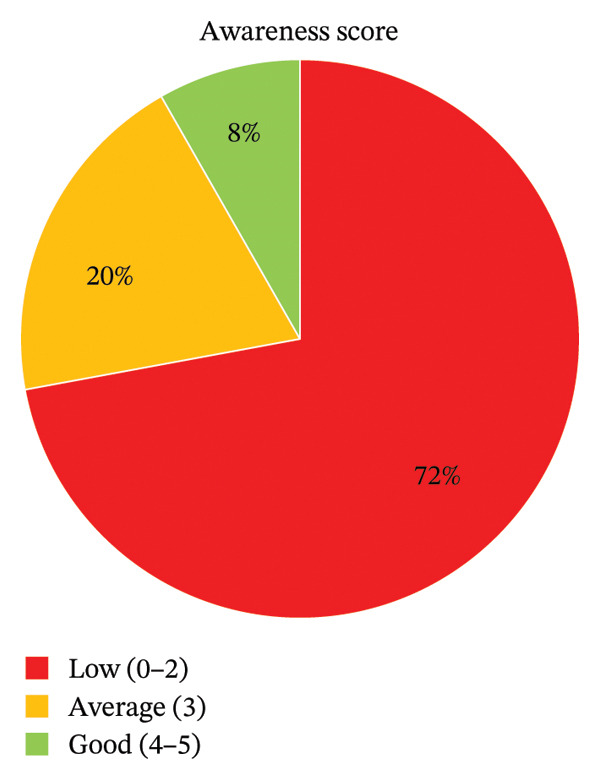
Awareness score (*n* = 384).

As shown in Table 3, awareness regarding safe medication disposal was poor. A significant majority (94.0%, *n* = 361) were unaware of drug take‐back systems, and most (88.0%, *n* = 338) had never seen instructions on how to dispose of medicines. Overall, 71.9% (*n* = 276) of participants were classified as having low awareness (Figure 1).

**TABLE 3:** Respondents’ awareness of the medication disposal’ (*n* = 384).

Variable	*n*	%
Knowledge of drug waste		
No	219	57.0
Yes	165	43.0
Knowledge of drug disposal instructions		
No	338	88.0
Yes	46	12.0
Knowledge of the drug return system?		
No	361	94.0
Yes	23	6.0
Knowledge of the risks of inappropriate use of antibiotic use		
Yes	232	60.4
No	152	39.6
Knowledge on the health and environmental risks associated with inappropriate disposal of medicines		
Yes	218	56.8
No	166	43.2

**FIGURE 2:**
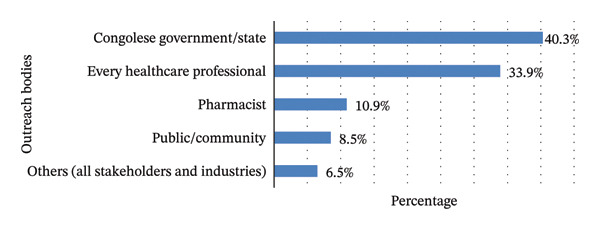
Participants’ views on bodies responsible for raising awareness of appropriate medicine disposal in the Boma Health Zone (*n* = 384).

Regarding responsibility for awareness, 40.3% (*n* = 155) of respondents cited the government and 33.9% (*n* = 130) cited healthcare personnel. Figure 2 illustrates these findings.

### 3.3. Attitudes Toward Medication Disposal

A substantial proportion (87.3%, *n* = 335) reported never receiving information on proper UEM disposal. This lack of awareness is further corroborated by strong agreement (62.3%, *n* = 239) that it contributes to the risks associated with UEM. Conversely, only a small minority (13.0%, *n* = 50) strongly disagree that healthcare providers adequately advise on disposal, suggesting a potential disconnect between perceived and actual practices.

**FIGURE 3:**
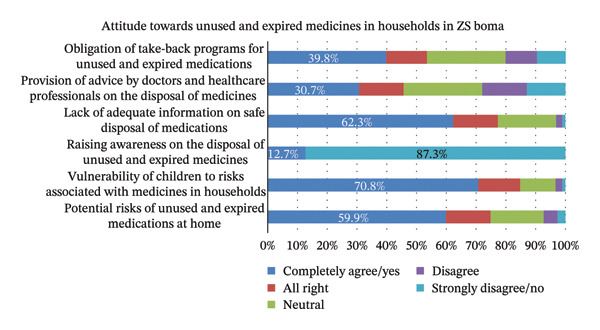
Attitude toward unused and expired medicines in households in ZS Boma (*n* = 384).

Encouragingly, a willingness to participate in take‐back programs is evident. Nearly 40% strongly agree that such programs should be mandatory, with a majority (53.5%) favoring mandatory implementation compared to those opposed (20.2%). A significant majority (nearly 60%) acknowledges the inherent risks of storing UEM in the home, particularly for children (70.8% strongly agree) (Figure 3).

**FIGURE 4:**
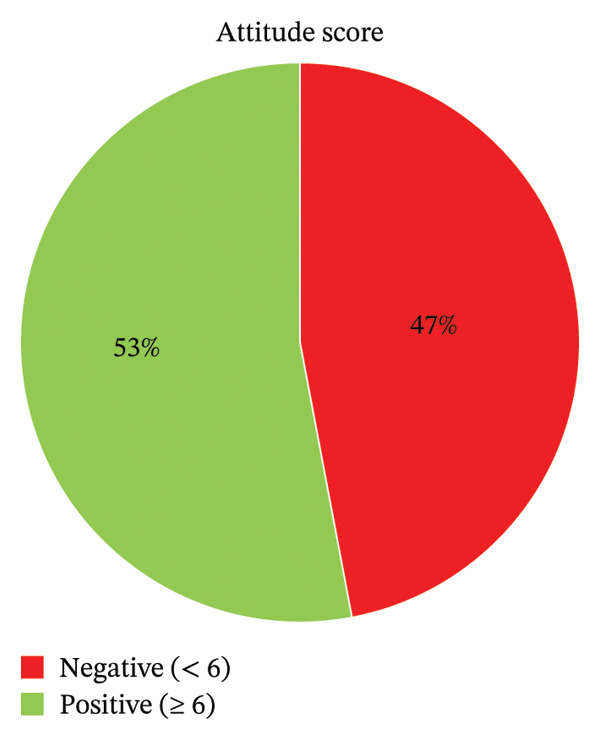
Attitude score (*n* = 384).

Analysis of medication possession revealed that the primary reason for possessing UEM was symptom resolution (70.6%, *n* = 271). Also, as potential strategies to minimize the accumulation of unused medications within the household, most respondents (56.5%, *n* = 217) suggested that appropriate advice be provided to the consumer (Table 4). Attitudinally, 53.4% (*n* = 205) of respondents were classified as having a positive attitude toward safe disposal (Figure 4).

**TABLE 4:** Reasons for possession and suggested actions to minimize accumulation; and attitude scores of respondents (*n* = 384).

	*n*	%
Reason for possession of unused medication		
Disease had disappeared	271	70.6
Adverse drug reactions	51	13.3
Change in treatment by healthcare professional	33	8.6
Professional prescribed more than necessary	15	3.9
Other	14	3.6
Actions to be taken to minimize or control the dangerous effect		
Providing appropriate advice to the consumer	217	56.5
Prescribing quantities and durations that guarantee patient compliance	84	21.9
Giving away unused drugs	41	10.7
Reducing the number of drugs prescribed by the doctor	41	10.7
Other	1	0.3
	0	0.0

### 3.4. Medication Disposal Practices

Over half (53.4%, *n* = 205) of respondents had UEM stored at home, most commonly analgesics and antibiotics (45.3%, *n* = 174). The disposal practices were predominantly unsafe (Table 5).

**TABLE 5:** Disposal practices for unused and expired medicines (*n* = 384).

	*n*	%
Do you have any unused or expired medication at home?		
Yes	205	53.4
No	179	46.6
Types of pharmaceutical products left unused at home		
Analgesics and antibiotics	174	45.3
Analgesics	101	26.3
Antibiotics	62	16.1
Antihypertensives	21	5.5
Other	21	5.5
Antidiabetics	6	1.6
Method for disposing of UNUSED medications		
Keep at home until expired	130	33.9
Dispose of in household waste	61	15.9
Burn	55	14.3
Give to friends or family	53	13.8
Return to pharmacy	30	7.8
Do not know	30	7.8
Donate to the hospital	19	4.9
Flush down toilet or sink	6	1.6
Practice of separating unused drugs before discarding of them		
No	332	86.5
Yes	52	13.5
Method for disposing of EXPIRED medications		
Burn	160	41.7
Dispose of in household waste	126	32.8
Bury/bury underground	37	9.6
Flush down toilet	36	9.4
Return to pharmacy	17	4.4
Do not know what to do	6	1.6
Other	2	0.5

For unused medicines, the most frequent approach was to keep them at home until expiry (33.9%, *n* = 130), followed by household garbage (15.9%, *n* = 61), burning (14.3%, *n* = 55), and giving them to family/friends (13.8%, *n* = 53).

**FIGURE 5:**
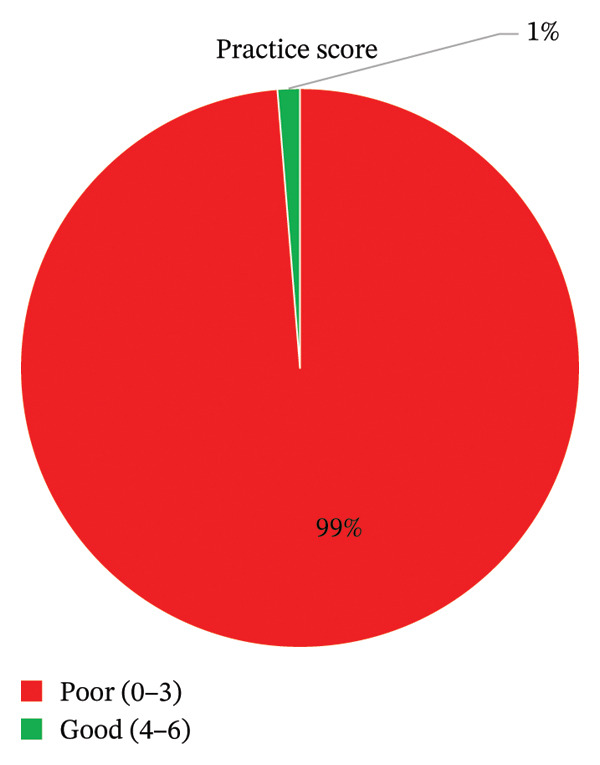
Practice score (*n* = 384).

The most common methods for disposing of expired medicines were burning (41.7%, *n* = 160) and throwing them in household garbage (32.8%, *n* = 126). Only 4.4% (*n* = 17) returned expired medicines to a pharmacy. Consequently, 98.7% (*n* = 379) of participants were classified as having poor disposal practices (Figure 5).

**FIGURE 6:**
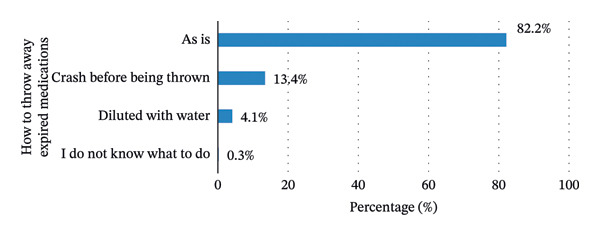
Reported practices for disposing of expired medicines (*n* = 384).

Figure 6 illustrates the methods for disposing of expired medications.

Over 80 percent of respondents (82.2%) threw away expired medicines as they are. 13.4% crushed them before throwing them away. and only 4.1% said that expired medicines were thrown away after dilution.

## 4. Discussion

This study provides new evidence on household awareness, attitude, and practice regarding UEM in the Boma HZ of the DRC. The findings reveal a major public health and environmental concern, shaped by poor awareness, unsafe practices, and the absence of formal waste management systems.

Only 6.0% of participants had ever heard of take‐back systems, and fewer than 12.0% had received any information on safe disposal. These figures are much lower than those reported in Ethiopia (66.9%–87.2%) [12, 13] or Europe (29%–43%) [20, 21]. This lack of awareness highlights systemic communication gaps between health providers and communities in Boma. However, the fact that more than half of respondents demonstrated a positive attitude suggests a strong potential for behavioral change if awareness programs are introduced.

Burning (41.7%) and discarding in household waste (32.8%) were the dominant methods for expired medicines. Open burning releases toxic pollutants and particulates into the air [6], while disposal in trash leads to contamination of soil and water, especially in a context like Boma, where waste management is informal and the Kalamu River is already known to be polluted [15, 16]. These practices align with findings from Ghana [11] and Bangladesh [22] but stand in stark contrast to the safer practices (e.g., pharmacy returns) common in countries with structured take‐back systems. The very low rate of pharmacy returns (4.4%) underscores the absence of such options for households in Boma.

The high prevalence of UEM storage (53.4%), particularly of analgesics and antibiotics, is concerning. This pattern, also seen in South Africa [14] and Pakistan [23], increases the risks of self‐medication, and accidental poisoning and contributes to the global threat of AMR. While 60.4% of respondents recognized the risk of antibiotic resistance, this awareness level is lower than in some Ethiopian studies [12, 13], suggesting a need for more targeted educational efforts in the DRC.

Only 12.8% of respondents had received information on appropriate disposal. This rate is higher than South Africa (4.7%) and Nigeria (6.4%) [10, 14] but lower than Ghana (19%) and the United States (20%) [11, 24]. This underscores the systemic failure of communication between healthcare providers and patients in Boma. Encouragingly, 53.4% of respondents demonstrated a positive attitude toward proper disposal, suggesting openness to change if provided with guidance.

The findings in Boma are consistent with broader challenges reported in Asia. In India, over 83% of respondents discarded UEM in household trash, and less than 5% used pharmacy returns [25]. In Bangladesh, most UEM was disposed of into drains or trash [22]. In Pakistan, 87% of households stockpiled medicines, with 69% disposing of them in trash or toilets and only 5.5% returning them to facilities [23]. In Vietnam, 70% of expired medications were thrown in household waste, with only 12.6% aware of take‐back systems [26]. These parallels highlight a regional and global pattern: in the absence of regulatory frameworks and public education, unsafe disposal becomes the default practice.

Together, these findings point to systemic gaps in pharmaceutical waste management in Boma. Unsafe practices endanger both public health and the environment, while the absence of policy support and structured programs perpetuates risky behaviors. At the same time, the positive attitudes observed represent an opportunity: the community is receptive to interventions, provided these are practical and supported by trusted actors such as pharmacists and community leaders.

### 4.1. Limitations

This study has limitations. First, reliance on self‐reported practices may have introduced social desirability bias, potentially overestimating positive behaviors. Second, the cross‐sectional design provides only a snapshot and cannot establish causal relationships between awareness and practices. Third, the study was geographically limited to the Boma HZ, and results may not be generalizable to rural or northern areas of the DRC with different sociocultural contexts. Finally, the absence of qualitative data restricted exploration of deeper motivations behind disposal practices.

### 4.2. Policy and System Implications

These findings point to systemic weaknesses in pharmaceutical waste management in Boma. At present, pharmacies are not integrated into waste collection, and there is no national regulatory framework. Introducing pharmacy‐based take‐back schemes, supported by government regulation and community engagement, would provide households with safer options. In the longer term, policies should align with WHO recommendations, including extended producer responsibility and investment in safe incineration facilities.

## 5. Conclusion and Recommendations

This study concludes that critical gaps in knowledge and unsafe disposal practices regarding UEM are pervasive among households in the Boma HZ. The widespread use of methods like open burning and trash disposal poses significant risks to environmental quality and public health, including the exacerbation of AMR. However, the identified positive attitudes and willingness to participate in take‐back programs present a valuable opportunity for intervention.

Based on these findings, we propose a coordinated, multilevel response:

1. Immediate Action (Community Awareness): Launch targeted awareness campaigns using local radio stations, religious institutions, and community health workers. Messages should focus on the risks of unsafe UEM disposal and promote interim safer practices (e.g., not flushing medications) while advocating for the development of formal systems.
2. Medium‐Term Strategy (Pilot Programs and Capacity Building): Establish and promote pilot take‐back programs in collaboration with community pharmacies in Boma. This should be accompanied by training for healthcare providers (doctors, pharmacists, and nurses) to counsel patients on proper disposal and the existence of take‐back points.
3. Long‐Term Policy (National Framework): Advocate for the development and implementation of a national regulatory framework for pharmaceutical waste management. This framework should mandate and fund take‐back systems, incorporate Extended Producer Responsibility, and outline standards for the safe transportation and destruction of collected UEM.

Future research should employ mixed‐methods approaches to better understand the behavioral drivers behind UEM disposal, conduct cost‐effectiveness analyses of take‐back pilot programs, and undertake longitudinal studies to measure the impact of interventions. Expanding this research to other provinces in the DRC will be crucial for developing a nationally relevant strategy.

By addressing this issue proactively, the DRC can mitigate a growing environmental health threat, protect its communities, and contribute to the global fight against AMR.

**Nomenclature**
UEM: Unused and expired medications
DRC: Democratic Republic of Congo
HZ: Health Zone
WHO: World Health Organization

## Funding

No funding was received for this manuscript.

## Conflicts of Interest

The authors declare no conflicts of interest.

## Data Availability

The data that support the findings of this study are available in the supporting information of this article.

## References

[1] Bound J. P. and Voulvoulis N. , Household Disposal of Pharmaceuticals as a Pathway for Aquatic Contamination in the United Kingdom, Health Perspectives. (2005) 113, no. 12, 1705–1711, 10.1289/ehp.8315, 2-s2.0-29144502284. PMC1314909 16330351

[2] Abahussain E. A. , Ball D. E. , and Matowe W. C. , Practice and Opinion Towards Disposal of Unused Medication in Kuwait, Medical Principles and Practice. (2006) 15, no. 5, 352–357, 10.1159/000094268, 2-s2.0-33746932876. 16888392

[3] Beirens T. M. J. , van Beeck E. F. , Dekker R. B. J. , and Raat H. , Unsafe Storage of Poisons in Homes With Toddlers, Accident Analysis & Prevention. (2006) 38, no. 4, 772–776, 10.1016/j.aap.2006.02.007, 2-s2.0-33646365042. 16545327

[4] World Health Organization , Addressing the Global Shortage of and Access to Medicines and Vaccines: Report by the Director-General [Internet], 2018.

[5] WHO-World Health Organization , Promoting Rational Use of Medicines: Core Components, 2002, WHO Policy Perspective Med, https://apps.who.int/iris/bitstream/handle/10665/67438/WHO_EDM_2002.3.pdf.

[6] Kronacher C. and Hogreve F. , Röntgenologische Skelettstudien an Dahlemer Binder‐Drillingen Und Zwillingen, Zeitschrift für Züchtung RB. Tierzüchtung und Züchtungsbiologie Einschließlich Tierernährung. (1936) 36, no. 3, 281–294.

[7] Benotti M. J. , Trenholm R. A. , Vanderford B. J. , Holady J. C. , Stanford B. D. , and Snyder S. A. , Pharmaceuticals and Endocrine Disrupting Compounds in US Drinking Water, Science and Technology. (2009) 43, no. 3, 597–603, 10.1021/es801845a, 2-s2.0-63149135884. 19244989

[8] Beere W. , Mullet S. , Wingstedt E. B. Ø. , Savoainen S. , and Lahti T. , Nuclear Plant Instrumentation, Control and Human-Machine Interface Technologies. International Topical Meeting. 7TH 2010., NPIC HMIT. (2010) 3, no. 2, 1920–1924.

[9] Seehusen D. A. and Edwards J. , Patient Practices and Beliefs Concerning Disposal of Medications, The Journal of the American Board of Family Medicine. (2006) 19, no. 6, 542–547, 10.3122/jabfm.19.6.542, 2-s2.0-33750965732. 17090787

[10] Adedeji-Adenola H. , Adesina A. , Obon M. et al., Knowledge. Perception and Practice of Pharmaceutical Waste Disposal Among the Public in Lagos State, Nigeria, Pan African Medical Journal. (2022) 42. 10.11604/pamj.2022.42.106.34529 PMC9392007 36034015

[11] Sasu S. , Kümmerer K. , and Kranert M. , Assessment of Pharmaceutical Waste Management at Selected Hospitals and Homes in Ghana, Waste Management & Research. (2012) 30, no. 6, 625–630, 10.1177/0734242x11423286, 2-s2.0-84862269473. 22081380

[12] Ayele Y. and Mamu M. , Assessment of Knowledge. Attitude and Practice Towards Disposal of Unused and Expired Pharmaceuticals Among Community in Harar City, Eastern Ethiopia, Journal of Pharmaceutical Policy and Practice. (2018) 11, no. 1, 1–7, 10.1186/s40545-018-0155-9, 2-s2.0-85056742770. 30459955 PMC6236888

[13] Kahsay H. , Ahmedin M. , Kebede B. , Gebrezihar K. , Araya H. , and Tesfay D. , Assessment of Knowledge, Attitude, and Disposal Practice of Unused and Expired Pharmaceuticals in Community of Adigrat City, Northern Ethiopia, Journal of Environmental and Public Health. (2020) 2020, 1–11, 10.1155/2020/6725423. PMC7178471 32351582

[14] Mahlaba K. , Helberg E. , Godman B. , Kurdi A. , and Meyer J. , Patients’ Knowledge and Practice on Disposal of Medicines Kept in Households in South Africa: Findings and Implications, Journal of Research in Pharmacy Practice. (2022) 11, no. 1, 13–18, 10.4103/jrpp.jrpp_85_21. 36277964 PMC9585805

[15] Congo D. R. , Congo Sciences, 2015.

[16] Simbu A. V. , Sanghy S. P. , Mulomba P. M. et al., Elements of Assessment of a Watershed With a View to a River Contract: Case of the Kalamu in Boma (Kongo Central. DR Congo).

[17] Edward L. , Democratic Republic of Congo Health Zones Map, 2020.

[18] CARG , A look at the Bas-Congo Province, 2011.

[19] Scientific Commission on War Crimes , The Nuremberg Code-1947, 1947.

[20] Vogler S. , Leopold C. , Zuidberg C. , and Habl C. , Medicines Discarded in Household Garbage: Analysis of a Pharmaceutical Waste Sample in Vienna, Journal of Pharmaceutical Policy and Practice. (2014) 7, no. 1, 1–8, 10.1186/2052-3211-7-6, 2-s2.0-84904768232. 25848546 PMC4366941

[21] Persson M. , Sabelström E. , and Gunnarsson B. , Handling of Unused Prescription Drugs-Knowledge, Behavior and Attitude Among Swedish People, Environment International. (2009) 35, no. 5, 771–774, 10.1016/j.envint.2008.10.002, 2-s2.0-67349161475. 19013646

[22] Islam S. , Public Awareness and Practices Regarding Pharmaceutical Waste in Urban Bangladesh, International Journal of Environmental Research and Public Health. (2019) 16, no. 8.

[23] Khan F. U. , Household Disposal of Unused Medications in Quetta, Pakistan: A Cross-Sectional Study, Journal of Applied Pharmaceutical Science. (2023) 13, no. 2, 10–15.

[24] Ruhoy I. S. and Daughton C. G. , Beyond the Medicine Cabinet: An Analysis of Where and Why Medications Accumulate, Environment International. (2008) 34, no. 8, 1157–1169, 10.1016/j.envint.2008.05.002, 2-s2.0-52349085845. 18571238

[25] Kaur H. , A Cross-Sectional Survey to Assess the Drug Disposal Practices of Unused and Expired Medicines Among Lay Public Visiting a Tertiary Care Hospital in Mumbai, India, International Journal of Pharmacy Practice. (2024) .

[26] Nguyen T. M. , Le V. L. , Nguyen T. M. et al., Urban Medication Disposal Practices in Vietnam: A Cross-Sectional Population Study, Nature Scientific Reports. (2025) 15, no. 1, 76–85, 10.3390/nano15010076.

